# Predation pressure on sentinel prey does not necessarily diminish with advancing urbanization

**DOI:** 10.1111/1744-7917.70151

**Published:** 2025-08-21

**Authors:** Gábor L. Lövei, Roland Horváth, Szabolcs Mizser, Mária Tóth, Tibor Magura

**Affiliations:** ^1^ HUN‐REN–UD Anthropocene Ecology Research Group Debrecen Hungary; ^2^ Department of Agroecology, Flakkebjerg Research Centre Aarhus University Slagelse Denmark; ^3^ Faculty of Science and Technology, Department of Ecology University of Debrecen Debrecen Hungary

**Keywords:** artificial caterpillars, predation, rural forests, seasonality, urban forests

## Abstract

Urbanization, one of the most significant global environmental issues of our time, causes significant environmental and structural changes in natural or seminatural habitat patches. These urbanization‐related changes trigger significant impact on ecological interactions and functioning. Predation is one of the most important ecological interactions, and urbanization‐related changes on predation pressure may have substantial ecological consequences. We studied predation pressure over a full season (from April to October) in rural versus urban forests using the sentinel approach in and around a large city (Debrecen) in the eastern part of the Great Hungarian Lowland. Model caterpillars made of nondrying green plasticine were readily attacked by arthropods, birds and mammals. From attack marks left by potential predators, a relatively high predation pressure was documented: up to 36% of the caterpillars exposed for 24 h showed attack marks. Seasonal differences were also obvious, with predation pressure during summer being significantly higher than in spring or autumn. This trend held for overall attack rates, also for attacks by arthropods and mammals but not birds. Surprisingly, attack rates were often higher in urban than rural habitats, contradicting the general hypothesis that predation pressure is lower in urbanized areas. As attack rates depend on both predator abundance and activity, and general data indicate lower predator abundances in urban habitats, this phenomenon may have been caused by hungrier predators in urban forest fragments or by the predator relaxation/safe habitat hypothesis that argues that a reduced need for vigilance allows more time to search for prey.

## Graphical abstract

Using artificial caterpillars as sentinels, we documented the predatory activity of arthropods, birds, and small mammals. Predator activity was highest during the summer and unexpectedly, significantly higher in urban than rural forest fragments. This may have been caused by hungrier predators, predator release, or safer urban habitat.

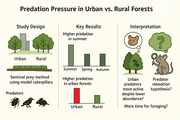

## Introduction

Predation is one of the most important ecological interactions, especially for heterotrophs: they cannot create their necessary resources from inorganic substances; therefore, they need to feed on other organisms, be those autotrophs or heterotrophs (Taylor, 1984). There is a multitude of predators both invertebrates and vertebrates, in all habitats and their effects are multiple: predation can create specific dynamics in ecological communities by eliminating more individuals from populations of certain species but not others, keep resources recycled more effectively than decomposition, influence nutrient cycling (Leroux & Schmitz, 2015) and even landscape composition via modifying the spatial distribution of the activity of big herbivore prey (Lima, 1998).

One of the evolutionarily newest types of habitats are those of human settlements. The proliferation of humans started only a few hundred years ago and critically accelerated in the second half of the 20th century (Shoshitaishvili, 2021). Today, urbanization is recognized as one of the fastest and most powerful agents of human‐induced global change (Nuissl & Siedentop, 2021). Urbanization has substantial effects at various levels of biological organizations through triggering profound changes in influential environmental factors (Theodorou, 2022). Urban areas are not necessarily devoid of biodiversity, but this diversity is profoundly modified in abundance and composition with respect to the biodiversity of the original habitat that the settlement now occupies (Soanes *et al.*, 2023). Urban‐living species react to the changed conditions at various levels, from changing in individual behaviour (Gervais *et al.*, 2025), modified microbiomes (Magura *et al.*, 2024), and alterations during the duration of individual development, manifested in fluctuating asymmetry (Elek *et al.*, 2017). There are changes at the assemblage level, causing the specialists, which usually have narrower tolerance limits (Devictor *et al.*, 2008), to decrease. Less is known about ecological processes, although soil‐living organisms demonstrably show changes in diversity and abundance (Szabó *et al.*, 2023).

A logical consequence of the substantial changes in biodiversity is to expect changes in ecological interactions and functioning. These, however, are less studied than changes in structural parameters in ecological assemblages. Thus, urban ecological studies more often examine changes in the composition of ecological communities rather than the functional consequences of these structural changes.

When contemplating the possible effect of urbanization on predation, arguments can be found that it is reduced but also that it intensifies, forming the “predation paradox” (Fischer *et al.*, 2012). The loss of large predators that humans do not tolerate around them, and the general disturbance caused by a high density of humans and their activities argue for reduced predation pressure: there are fewer predators and more human disturbance that decreases predator activity. However, human settlements also concentrate resources, so there can be a lot of available resources, often increasing the abundance of potential prey. For example, birds in urban areas have lower diversity but increased abundance (Ferrarini *et al.*, 2025). This could boost the abundance of predators that can tolerate the human‐modified environment. There can also be a mesopredator release due to the absence of large predators (Soulé *et al.*, 1988). For example, wolves and leopards often limit the density of smaller predators such as foxes and cats. The absence of the former can allow an increase in the densities of smaller predators, causing higher predation pressure on their prey. A similar argument can be extended to invertivorous predators: fewer owls mean more ground beetles, for example, and thus a higher potential predation pressure on their small invertebrate prey.

A traditional approach to study urbanization effects is by the gradient approach (Seress *et al.*, 2014). However, the gradient is sometimes presented as an urban‐rural gradient—which is the wrong direction, as argued by Magura *et al.* (2010), because that misidentifies the base of comparison, triggering a misbelief that “things are improving.” In reality, the question is “how much can be preserved with respect to the original situation.” And that means that the baseline is the rural situation, and the proper direction of comparison is rural‐urban. Additionally, the gradient itself is often too loosely defined, especially due to the lack of well‐articulated criteria identifying the suburban stage. The structure of suburbia can be extremely variable due to socioeconomic and cultural reasons (Seress *et al.*, 2014).

We aimed to test the changes in predation pressure on insects in an eastern European city (Debrecen) using the sentinel approach (Howe *et al.*, 2009). Specifically, we formulated the following hypotheses:

**H1**: Predation pressure will be lower in urban areas than the respective rural habitat. This would be a logical consequence of the generally decreased abundance and diversity of invertebrates in urbanized areas (Svenningsen *et al.*, 2024).
**H2**: We also hypothesized seasonal differences in predation pressure, with maximum levels in summer when the prey base is expected to be the highest, due to the presence of immature developmental stages such as eggs and larvae.
**H3**: We also hypothesized that the main groups of predators would display different seasonal dynamics. Bird predation pressure is expected highest during late spring, when adults feed their nestlings (Zvereva & Kozlov, 2021), while the highest invertebrate predation was expected in summer (Eötvös *et al.*, 2020).

Our results supported H2 and H3 but contradicted H1: the recorded predation pressure was highest during summer, but such pressure was higher in urban than rural areas.

## Materials and methods

### Study area and sampling

We followed the approach of the GLOBENET (Global network for assessing the impacts of landscape change on biodiversity) project (Niemelä *et al.*, 2000) that constrains the rural‐urban gradient the following way: (i) the original rural habitat has to be forest, preferably under light management and no history of clear‐cutting; and (ii) the urban habitats have to be documented remnants of this original forest (that still exists outside the urban area), even if modified by later planting of trees or bushes, and under park management.

Such a setup exists in the eastern Hungarian city of Debrecen which is famous for its former “Big Forest” that has been preserved on the otherwise scarcely forested Great Hungarian Plain. Over the 19th−20th centuries, the city started to encroach on this forest, creating a patchwork of parks and forest fragments that originally belonged to the city forest (Magura *et al.*, 2018). However, due to the uncertainties concerning the definition of suburban stage, we only considered the two ends of the spectrum: the rural versus the urban habitats.

We selected a total of 8 oak‐dominated forest sites, 4 in the rural and 4 in the urban areas [details described in Magura *et al.* (2024); see also https://data.mendeley.com/datasets/ptmzyr74zx/1 for a KLM file showing the location of the sites]. In every site, we deployed 20−20 artificial caterpillars (20 mm long, 3 mm thick, glued with Loctite Power Flex superglue onto a 50 mm × 30 mm bark piece; made of light green plasticine, Smeedi plus, V. nr. 776609, Vilborg, Denmark from April to October 2021, 2020). At the center of all 8 sites, we marked a 25 m × 25 m square, over which we laid a 5 m × 5 m raster. Within the 36 nodes thus created, in each month we identified 20 random positions where caterpillars were deployed.

The caterpillars were prepared as suggested by Howe *et al.* (2009) and exposed for 24 h at ground level. Of the total of 1120 caterpillars exposed (8 areas × 7 sessions × 20 caterpillars each), 13 caterpillars (1.2%) went missing or was damaged/destroyed; these were excluded from the evaluation. On collection, each individually identified caterpillar was put into an Eppendorf tube, taken to the laboratory and examined under a stereomicroscope (3−20× magnification) for attack marks. Occasionally, we found multiple attack marks by the same type of predator (i.e., a bird). In order to avoid inflating the attack probability, these cases were registered as a single attack, unless the marks were clearly made by different predators (different shape or attack angle). Predators were classified from their characteristic marks attributable to arthropods, birds, and small mammals (Low *et al.*, 2014). The period of April and May was considered spring, June–August as summer, and September−October as autumn. Predation rate was quantified as the proportion of caterpillars attacked (Eötvös *et al.*, 2020).

### Statistical analyses

All statistical analyses were performed using the R program environment (version 4.4.1; R Core Team, 2024). Differences in predation rate between the rural and urban habitats were tested using generalized linear mixed model (GLMM) with the help of the *lme4* package (Bates *et al.*, 2015). Before modeling, the probability distribution that best fitted the response variable was examined by the *MASS* package (Venables & Ripley, 2002). Based on these analyses, all response variables (overall predation rate, mammalian predation rate, bird predation rate, arthropod predation rate) were modeled using lognormal error distribution. In the models the fixed effects were the urbanization level (rural vs. urban), the season (spring, summer, autumn), as well as their interaction. The nested nature of our sampling design (sites nested within areas), as well as the sampling month was considered a random effect. As the variables fitted a lognormal distribution, the parameters in the mixed models were estimated using the Penalized Quasi‐Likelihood (PQL) method (Breslow & Clayton, 1993; Zuur *et al.*, 2009). In case the GLMMs revealed a significant difference, we performed multiple comparisons among means using the *agricolae* package (de Mendiburu, 2023).

## Results

### Overall attack rates

Mean total attack rates was 19.9%d^−1^ (12.7%d^−1^ by arthropods, 2.2%d^−1^ by birds, and 6.1%d^−1^ by mammals). Overall attack rates, grouped by season and habitat, varied between 7.8%d^−1^ (rural habitat, autumn) and 36.4%d^−1^ (urban habitat, summer, see Table 1). Attacks by arthropods were more frequent (maximum 25.3%d^−1^, urban habitats, summer), sometimes by an order of magnitude higher, than attacks by birds (maximum 2.9%d^−1^ rural, summer) or mammals (maximum 11.1%d^−1^, urban, summer).

**Table 1:** Seasonal attack rates on artificial caterpillars in rural and urban forests by the main types of predators near the city of Debrecen, Hungary

		Attack rates (%d^−1^) by				
Season	Habitat	All predators	Arthropods	Birds	Mammals	Sample size^†^
Spring	Rural	8.75 (5.8)	5.63 (4.2)	2.50 (3.8)	0.63 (1.8)	8
	Urban	10.76 (8.8)	8.82 (5.8)	0.63 (1.8)	3.82 (5.9)	8
Summer	Rural	24.76 (11.2)	13.46 (8.6)	2.94 (3.4)	9.19 (12.6)	12
	Urban	36.44 (21.8)	25.31 (21.9)	2.54 (3.4)	11.13 (11.0)	12
Autumn	Rural	7.76 (7.9)	3.26 (5.6)	2.60 (4.0)	1.91 (3.8)	8
	Urban	20.03 (11.3)	13.13 (11.6)	1.25 (2.3)	5.66 (6.2)	8

^†^ Sample size equals the number of monthly sessions × habitat patches.

Note: Numbers are means; SD values are in parentheses.

### Attack rates in rural versus urban habitats

Overall attack rates over the full season were significantly higher in urban than rural habitats (Table 2). This pattern was likely to be driven by arthropod predation, as predation rates by arthropods were also significantly higher in urban than in rural habitats, while there was no similar difference in predation rates by mammals or birds (Table 2).

**Table 2:** Analysis of deviance table for the fitted generalized linear mixed model on overall predation rate, as well as on attack rates by the main types of predators (mammals, birds, and arthropods) in forested rural and urban habitats over a full season (from April to October) in and near the city of Debrecen, Hungary (*P* values in bold denote significant effects, *P* < 0.05)

Response variable	Explanatory variable	*χ* ^2^	*df*	*P*	Result of the LSD test
Overall predation rate	Habitat	5.1580	1	**0.0231**	Urban > Rural
	Season	35.3784	2	**< 0.001**	Summer > Spring ≈ Autumn
	Habitat × Season	1.6065	2	0.4479	
Mammal predation rate	Habitat	0.7398	1	0.3897	
	Season	14.6063	2	**0.0007**	Summer > Spring ≈ Autumn
	Habitat × Season	0.2012	2	0.9043	
Bird predation rate	Habitat	1.7789	1	0.1823	
	Season	1.5714	2	0.4558	
	Habitat × Season	0.6163	2	0.7348	
Arthropod predation rate	Habitat	8.1851	1	**0.0042**	Urban > Rural
	Season	14.0817	2	**0.0009**	Summer > Spring ≈ Autumn
	Habitat × Season	1.1290	2	0.5686	

Note: Results of the LSD test indicate which habitat/season differed significantly (*P* < 0.05) from others.

### Seasonal changes in attack rates

**Fig. 1:**
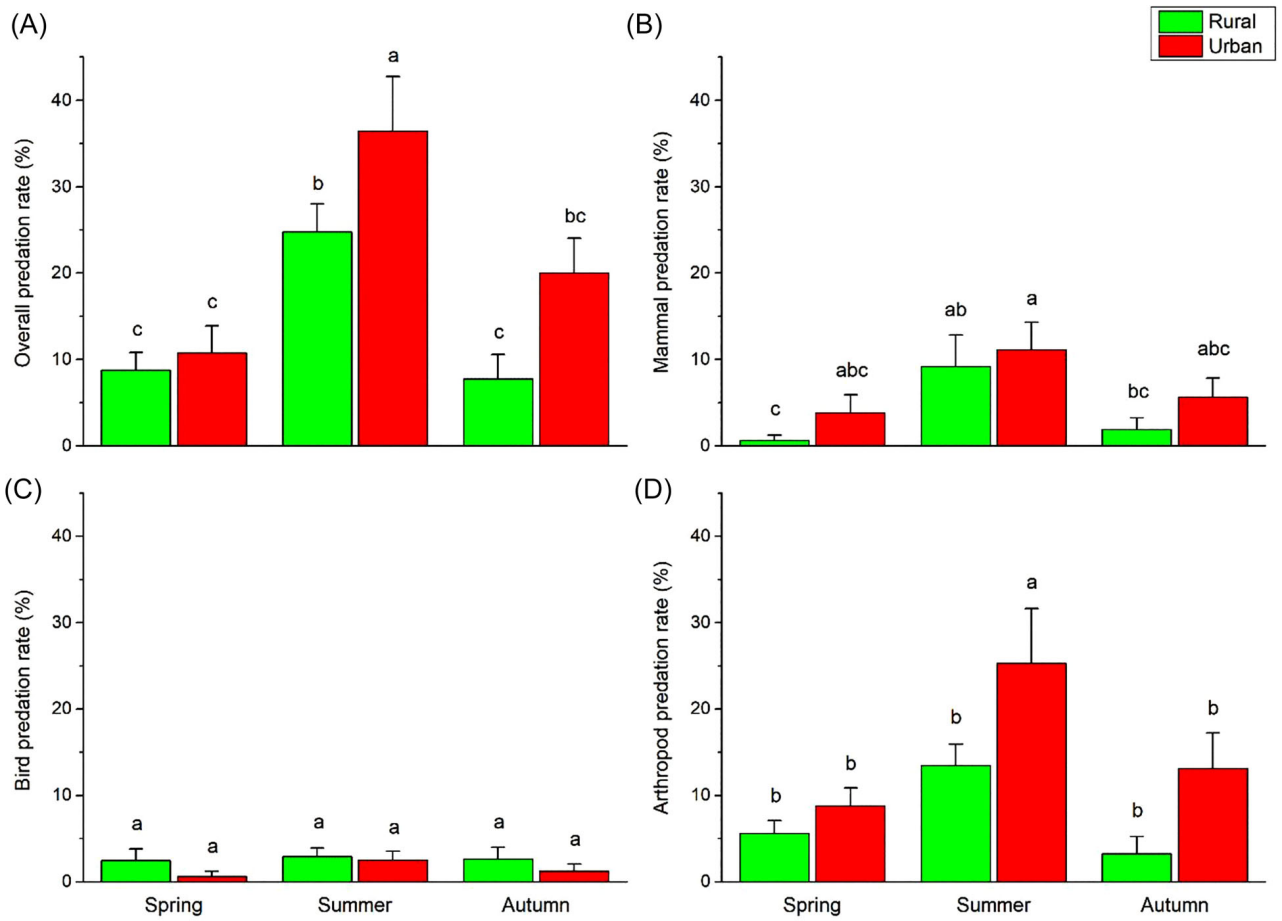
Overall (A), mammal (B), bird (C), and arthropod (D) predation rate (mean ± SE) on artificial caterpillars in rural and urban forested habitats over a full season (from April to October) in and near the city of Debrecen, Hungary. Different letters above the means indicate significant (*P* < 0.05) differences based on the LSD test.

Attack rates were significantly different by season for overall, mammal and arthropod attacks but not for those by birds. In all those cases, spring and autumn rates were not different, but significantly more attacks occurred during the summer months (Table 2 and Fig. 1).

For overall predation rates, there were no differences in seasonal trends in the two habitat types: summer values were significantly higher than those observed in spring or autumn both in rural and urban habitats, with no significant difference between spring and autumn. For birds, seasonal predation rates were not significantly different in either habitat. However, for mammal and arthropod attacks, the seasonal trends were slightly different. Mammal predation pressure in rural habitats was significantly higher in summer than in spring, while no similar difference was registered in urban habitats. On the contrary, in urban habitats the arthropod attacks were significantly the highest in summer, while in rural habitats attack rates were not significantly different by season (Table 1 and Fig. 1).

## Discussion

### Predation pressure in rural versus urban habitats

Our results confirm that invertebrates are under considerable predation pressure: even our “nonrealistic” sentinel prey suffered a “mortality” of up to 40%d^−1^, mostly from arthropod predators. This is in the range documented earlier (Lövei & Ferrante, 2017). We did not find support for our hypothesis that urbanized habitats have decreased predation pressure: surprisingly, a higher predation pressure was registered in urban than in rural forests for most predator groups, especially for arthropods. In a meta‐analysis, Eötvös *et al.* (2018) documented a significant decrease in predation pressure with advancing urbanization. It has to be noted, however, that this was based on only 35 studies, most of them being studies of predation on bird nests. In that review, only one study (Ferrante *et al.*, 2014) considered invertebrate predation. These authors, using artificial caterpillars in the Danish location of the GLOBENET project also documented a decrease in predation pressure with advancing urbanization in Denmark (Ferrante *et al.*, 2014). To the best of our knowledge, following the publication of the comprehensive study of Eötvös *et al.* (2018), only one additional investigation has compared predation pressure on sentinel caterpillars exposed at ground level, also showing a decreasing predation pressure from rural to urban habitats (Eötvös *et al.*, 2020).

Our results, however, did not conform to these results. Our contradictory results can be interpreted by the predation‐paradox, emphasizing that predation intensity can be influenced by various factors and that the cumulative effect of urbanization on predation rate is often non‐uniform (Fischer *et al.*, 2012). Predation pressure can be lower (predation relaxation or safe habitat hypothesis, Gering & Blair, 1999) or higher in urban than rural habitats (predator proliferation hypothesis, Fischer *et al.*, 2012; mesopredator release, Prugh *et al.*, 2009). In the two above studies using sentinel insect prey, other factors not directly related to urbanization may also have influenced predation pressure. In the Danish study, the urban forest patches examined were located in a public park, mainly with open‐habitats (Ferrante *et al.*, 2014). The isolation of these forest fragments may have limited the movement of flightless, forest‐associated predators between patches, thereby reducing their within‐patch density and potentially decreasing predation pressure. Our urban sites were less isolated than those in the Danish study and were not influenced by other, not urbanization‐related disturbances. In the other study, floodplain forest stands were studied along an urbanization gradient (Eötvös *et al.*, 2020). Flooding events affecting the forest stands may have contributed to alterations in the predator communities inhabiting these patches, thereby influencing predation pressure. These facts highlight the importance of accounting for a range of factors−such as landscape‐level effects and other disturbances not directly associated with urbanization−when assessing the impact of urbanization on predation pressure. Furthermore, colder winter temperature is a limiting factor for small ectotherms, such as insects (Parsons & Frank, 2019; Bujan *et al.*, 2024). The urban heat island effect (Lövei & Magura, 2022) raises winter temperatures, increasing the success of insect overwintering, thereby intensifying predation pressure in urban habitats.

### Seasonal changes in predation pressure

In accordance with our hypothesis, attack rates were significantly higher in summer for overall, mammal and arthropod attacks. Similarly to our results, a previous study also reported higher attack rates of ground‐level predators on sentinel insect preys during summer (Eötvös *et al.*, 2020), suggesting increased prey availability (simultaneous presence of mature and immature prey) and/or increased predator activity, including juvenile predators. However, ambient temperature can affect the detectability of predation marks, so it is concluded that model caterpillars made of plasticine are not suitable for comparing predation pressure in habitats with significantly different temperatures, such as those along elevation or latitude gradients (Muchula *et al.*, 2019). The temperature of our studied habitats did not differ markedly, so it is unlikely that the higher predation pressure observed in summer was caused solely by the ambient temperature.

For mammal and arthropod predation pressure, the seasonal trends were slightly different in rural versus urban habitats. In rural habitats attack rates by mammal predators were significantly higher in summer than spring, while in urban habitats attack rates were not significantly different over the seasons. This may have occurred due to the impoverishment of small mammal assemblages in urban habitats (Gomes *et al.*, 2011) or due to a higher activity of mammals during all season, because of higher temperatures or more resources. The recorded lower attack rates of arthropods in spring and autumn in urban but not in rural habitats allow the plausible interpretation that the urban habitats can be sinks for invertebrate predators. An important and abundant group of invertebrate, ground‐active predators are carabid beetles. Several carabid species complete their individual development in late spring or early summer (spring‐breeders, Lövei & Sunderland, 1996) when young adults may start dispersing and invading other habitat patches. Such a phenomenon is documented for *Nebria brevicollis* in Denmark (Lövei *et al.*, 2018). Alternatively, due to the urban heat island effect (Lövei & Magura, 2022), summer heatwaves in urban areas result in a reduced availability of potential prey, as many prey species attempt to cope with the unfavorable hot and dry conditions by aestivation or emigration. This prey shortage in urban environments during summer may intensify predation pressure exerted by food‐deprived predators.

As observed in previous studies (Ferrante *et al.*, 2014; Eötvös *et al.*, 2020), frequency of attack marks by birds on sentinel caterpillars placed on the ground level was also quite low. Similarly, low bird predation pressure occurs on tree trunks (Eötvös *et al.*, 2020). Bird predation rate on artificial plasticine larvae deposited on tree branches, however, is notably high in the summer months, ranging from 25%−75%, and significantly higher in urban than rural situations (Kozlov *et al.*, 2017). These patterns highlight that prey selection, predation strategies, and predation pressure by insectivorous birds can differ significantly between ground‐level and canopy forager species. Therefore, to reliably quantify overall predation pressure in an ecosystem, members of various guilds should be simultaneously assessed.

### Methodological challenges

Given that predation on and by invertebrates is difficult to observe, and requires technologically challenging methods to prove, various approaches have been used (Lövei & Ferrante, 2024). Several of these are destructive which gradually makes them less acceptable on ethical grounds (Lövei *et al.*, 2023). Ecology has a well‐developed toolkit to collect, describe and analyze structural parameters of ecological communities (Henderson & Southwood, 2016) from which we try to deduce consequences for functioning. In many cases, measuring function intensity itself is simpler, nondestructive and provides sufficient information (Zvereva & Kozlov, 2023; Lövei & Ferrante, 2024). The sentinel prey method is one of these, and has been in use since the late 1970s (Lövei & Ferrante, 2017).

Sentinel prey can be real or artificial. All of them, be it a plasticine caterpillar, an immobilized live prey or a dead one, offer various cues to a potential consumer, some of which are real, others artificial. The advantage of the plasticine in comparison to more “realistic” sentinels is that we gain information about the attacker: the plasticine keeps the marks made by the attacker that can be identified to various levels of resolution (Low *et al.*, 2014). As with all similar methods where the studied organism's activity is necessary to generate a record, there are two possible components that influence the parameter in question: the abundance of the study subject, and their activity. It is impossible to separate the impact of the two components. In our case, a certain attack rate AR could be generated by an abundance Z of predators that display an activity level of Y (AR = Z × Y). A twice higher attack rate, 2AR could be achieved in two ways: either by an abundance of 2Z at an activity level of Y: (2AR = 2Z × Y), or by an unchanged abundance but twice higher activity levels (2AR = Z × 2Y). Consequently, we cannot tell whether there were more predators in the urban forest remnants, or just hungrier.

Previous studies on carabid assemblages in the same area (Magura *et al.*, 2004) documented that one clear effect of urbanization is the decrease in carabid abundance. The same seems to be a general effect on carabids due to urbanization (Martinson & Raupp, 2013). Carabids are one of the main predators of caterpillars, and there often is a correlation between carabid abundance and attack rates (e.g. Mansion‐Vaquié *et al.*, 2017)—but not in our studied habitats: here we have reduced carabid abundance but higher attack rates on the artificial caterpillars. We are also unaware of the nutritional status of the predators in the original forest stands versus its urban remnants. Predators have nutritional targets that are not easily satisfied, and if they find themselves at an imbalance, they will eat more to approach their optimal target (Jensen *et al.*, 2012). This might indicate suboptimal conditions in urban fragments in our studied city.

## Conclusions

It is increasingly clear that urban habitats provide benefits for their human inhabitants, and that these benefits are more satisfactory if the ecological procedures supporting these services are running as close to their original intensity as possible. In this respect our results seem reassuring: predation intensity may not necessarily be harmed by urbanization. However, the seasonal dynamics and possibly also the higher attack rates on the artificial caterpillars hint at adverse conditions in those fragments. The lower attack rates in spring might indicate lower overwintering success rates for invertebrates in urban habitats, and the higher rates later could be indications of higher level of hunger by predators than experienced by their rural conspecifics.

## Disclosure

The authors state that they have no conflict of interest.
